# De Novo Deletion of Chromosome 20q13.33 in a Patient with Tracheo-esophageal Fistula, Cardiac Defects and Genitourinary Anomalies Implicates GTPBP5 as a Candidate Gene

**DOI:** 10.1002/bdra.20821

**Published:** 2011-09

**Authors:** Benjamin D Solomon, Daniel E Pineda–Alvarez, Donald W Hadley, Amelia A Keaton, Nneamaka B Agochukwu, Manu S Raam, Hannah E Carlson–Donohoe, Aparna Kamat, Settara C Chandrasekharappa

**Affiliations:** 1 Medical Genetics Branch, National Human Genome Research Institute, National Institutes of HealthBethesda, Maryland; 2 Social and Behavioral Research Branch, National Human Genome Research Institute, National Institutes of HealthBethesda, Maryland; 3 HHMI–NIH Research Scholars Program, Howard Hughes Medical InstituteChevy Chase, Maryland; 4 Cancer Genetics Branch, National Human Genome Research Institute, National Institutes of HealthBethesda, Maryland

**Keywords:** tracheo-esophageal fistula, TE fistula, VACTERL association, 20q13.33 deletion, *GTPBP5*

## Abstract

**BACKGROUND:**

Tracheo-esophageal fistula (TEF) with/or without esophageal atresia (EA) is a common congenital malformation that is often accompanied by other anomalies. The causes of this condition are thought to be heterogeneous but are overall not well understood.

**CASE REPORT:**

We identified a patient with a TEF/EA, as well as cardiac and genitourinary anomalies, who was found to have a 0.7 Mb de novo deletion of chromosome 20q13.33. One gene within the deleted interval, *GTPBP5*, is of particular interest as a candidate gene.

**CONCLUSIONS:**

*GTPBP5* bears further study as a cause of TEF/EA accompanied by other malformations. Birth Defects Research (Part A) 2011. © 2011 Wiley-Liss, Inc.

## INTRODUCTION

Tracheo-esophageal fistula (TEF) with or without esophageal atresia (EA) is a relatively common malformation, occurring in approximately 1 in 3500 births (Torfs et al.,^1995^). Approximately half of patients have accompanying malformations, such as occur in Feingold syndrome, anophthalmia-esophageal-genital syndrome, CHARGE syndrome, vertebral anomalies, anal atresia, cardiovascular anomalies, tracheoesophageal fistula, esophageal atresia, renal or radial anomalies, or limb defects (VACTERL) association, and other, less well-characterized conditions involving multiple malformations. Among these accompanying congenital anomalies, cardiac defects are especially common (reviewed in Shaw–Smith,^2006^; de Jong et al., 2010).

Although the causes of some of the syndromic conditions that may include TEF/EA have been defined, the etiologies of others are less well-understood. TEF/EA is viewed as a complex, heterogeneous disorder resulting from multiple interacting genetic and environmental factors. Many questions remain regarding the genetic contributions to isolated TEF/EA, although there are hints from biologic models implicating key pathways including the Sonic hedgehog signaling network (reviewed in de Jong et al., 2010).

Specific chromosomal anomalies, such as trisomy 13, 18, and 21 have long been known to be associated withTEF/EA, as well as with other congenital malformations (Torfs et al.,^1995^). Chromosomal rearrangements visible on routine karyotype, as well as submicroscopic pathogenic copy number variations (CNVs) of numerous chromosomal loci, have also been implicated as causing TEF/EA. Some of these genomic imbalances have been reported in multiple affected individuals, while others have been described in only a single patient (Felix et al.,^2007^). Overall, these results support a heterogeneous model of causation.

We performed high-density microarrays on a cohort of 20 patients ascertained through our study on VACTERL association, with the hypothesis that this testing modality would reveal potentially explanatory genomic imbalances in some patients. One patient had a submicroscopic de novo deletion of chromosome 20q13.33, part of which did not overlap known regions of copy-number variation. Of note, while this patient met inclusion criteria for our study, most clinicians and researchers would not judge him as having “classic” VACTERL association. However, the deletion found in this patient suggests *GTPBP5* as a key gene contributing to the patient's presentation.

## CASE REPORT

### Patient

The patient was a 6-month-old white infant with a history of a TEF and EA, cardiac anomalies consisting of two ventricular septal defects, hypospadias, and a large unilateral hydrocele, as well as a large left cystic hygroma that self-resolved during infancy. He and his parents participated in our Institutional Review Board-approved National Human Genome Research Protocol on VACTERL association, with appropriate consent obtained from all participants. (Most clinicians and researchers would not consider this patient to have classic VACTERL association, as he did not have at least three component features, but he met the criteria for inclusion in our study based on the presence of two component features and an additional congenital malformation (Solomon et al.,2010a).

On physical examination, weight and height were slightly less than the third centile for age; head circumference was approximately 25th centile for age. He was not dysmorphic, and developmental milestones were appropriate at the time of evaluation, although at follow-up approximately 1 year later, he was reported to be receiving treatment for speech delay. He was his parents' only child; family history was noncontributory for any similar conditions. Traditional karyotype performed before research participation showed a normal male chromosome complement.

### Microarray Analysis

We extracted genomic DNA from peripheral blood samples using a QIAamp DNA Blood Maxi kit (Qiagen, Germantown, MD) per protocol. Microarray analysis was performed using the Illumina Omni1-Quad single-nucleotide polymorphism (SNP) array, which contains over 1 million SNP loci, with 300 ng of DNA (4 μl of 75 ng/μl DNA) per the Illumina “infinium assay” protocol (Gunderson et al.,^2005^). We collected data using the BeadArray scanner, and visualized data with the GenomeStudio (v2009.2, www.Illumina.com) genotyping module, using human genome build 36.1 (NCBI36/hg18) for analysis. The call rates for all the DNA samples were >99%. CNVs were detected using PennCNV software filtered to annotate regions with at least three contiguous SNPs with the same abnormality (Wang et al.,^2007^). Detected regions of genomic imbalances were compared to known CNVs in control populations with the Database of Genomic Variants (Zhang et al.,^2006^). Potentially pathogenic variants were further compared to 745 total individuals, none of whom had similar disorders, studied using high-density microarray platforms (192: Illumina OmniExpress; 206: Illumina 1M-Duo; 347: Illumina Omni1-Quad).

**Figure 1:**
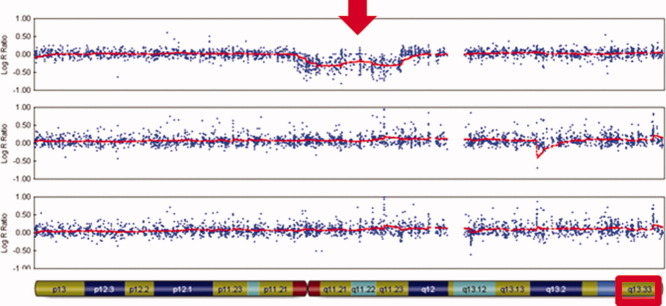
De novo 0.7 Mb deletion of chromosome 20q13.33 in the patient described here, ascertained with Illumina Omni1-Quad high-density single-nucleotide polymorphism (SNP) microarray platform, and visualized using GenomeStudio (v2009.2, www.Illumina.com) genotyping module. The deletion is indicated by the *arrow*; the corresponding chromosomal region is outlined in the ideogram below. The proband's microarray is uppermost; parental arrays of the same region are shown below the proband's. [Color figure can be viewed in the online issue, which is available at wileyonlinelibrary.com.]

The microarray revealed an approximately 0.7 Mb de novo (with parentage confirmed by microarray analysis) interstitial deletion on chromosome 20q13.33: arr20q13.33(59,671,821–60,329,092)x1 dn (Fig. 1). This deletion was confirmed by oligonucleotide microarray (targeted 60K oligonucleotide microarray performed at GeneDx, Gaithersburg, MD), as well as quantitative PCR of one of the genes (*TAF4*) in the deleted interval. Genes in the deleted interval include: *CDH4*, *MIR1257*, *TAF4*, *LSM14B*, *PSMA7*, *SS18L1*, *GTPBP5, HRH3, OSBPL2, LAMA5,* and *ADRM1*. There are small copy number variations in normal controls within this interval on chromosome 20, but none overlapping all of these genes or nearly as large as this deletion. In particular, deletions of *GTPBP5* have not been reported in control populations (Zhang et al.,^2006^). CNVs involving *LSM14B* have also not been reported, but very little is reported regarding the role of this gene. This deletion was additionally not found in 745 individuals who underwent high-density microarray analysis for reasons other than similar congenital malformations.

## DISCUSSION

Many different genomic imbalances have been reported in patients with TEF/EA (Felix et al.,^2007^). The deletion found in this patient is especially interesting as it suggests only two specific genes as being contributory: among the genes in the patient's deleted region, *GTPBP5* and LSM14B are the only ones that have not been reported as copy number variations in normal controls. Relatively little is known about the function of these genes, but interestingly, disturbances of the GTPBP5's function result in abnormal mitochondria (Hirano et al.,^2006^). Mitochondrial dysfunction is a well-documented link to congenital malformations such as those seen in VACTERL association, which this patient has features of, even if he were not deemed to meet strict diagnostic criteria (Damian et al.,^1996^; von Kleist–Retzow et al.,^2003^; Thauvin–Robinet et al.,^2006^; Solomon et al.,^2011^).

From a developmental standpoint, *LAMA5* is another interesting candidate gene, although with some strong caveats. *LAMA5*-null mice die in utero with multiple malformations; hypomorphic animals display anomalies such as renal and pulmonary malformations (Nguyen et al.,^2002^; Shannon et al.,^2006^). Further, *LAMA5* interacts with Sonic Hedgehog, a pathway frequently implicated in conditions that include TEF/EA such as VACTERL association (Kim et al.,^2001^; Gao et al.,^2008^). However, the mouse models do not correlate well with the specific malformations seen in this particular patient, and further, deletions of this gene have been reported in normal controls (Jakobsson et al.,^2008^).

The deleted region overlaps that reported in another patient, although this previously reported patient had a much larger deletion, making further comparisons quite speculative. This patient, like the one described here, was nondysmorphic; her phenotype interestingly included cardiac anomalies, as well as hydronephrosis (Traylor et al.,^2010^).

The fact that the deletion is de novo offers some evidence of possible pathogenicity. One additional possibility that cannot be ignored involves a recessive model in which the patient had, for example, a deletion of one allele and a point mutation in the other. However, further studies would be required to investigate this possibility.

Finally, it must be emphasized that while the patient was ascertained through a study focusing on VACTERL association, he had only two strict component features of VACTERL association, and thus should not be considered to have this diagnosis (although he also had genitourinary anomalies, which are frequent in patients with VACTERL association; Solomon et al.,2010b). Nevertheless, we hope that these results may be extrapolated to inspire further research on the causes of TEF/EA through molecular studies of *GTPBP5*.
